# Feasibility of ultraviolet light-emitting diode irradiation robot for terminal decontamination of coronavirus disease 2019 (COVID-19) patient rooms

**DOI:** 10.1017/ice.2021.95

**Published:** 2021-03-09

**Authors:** Hee Kyoung Choi, Chunguang Cui, Hyeri Seok, Joon-Yong Bae, Ji Hoon Jeon, Gee Eun Lee, Won Suk Choi, Man-Seong Park, Dae Won Park

**Affiliations:** 1 Division of Infectious Diseases, Department of Internal Medicine, Korea University Ansan Hospital, Ansan, Republic of Korea; 2 Department of Microbiology, Institute for Viral Diseases, College of Medicine, Korea University, Seoul, Republic of Korea

## Abstract

**Objective:**

To investigate the feasibility of using an ultraviolet light-emitting diode (UV LED) robot for the terminal decontamination of coronavirus disease 2019 (COVID-19) patient rooms.

**Methods:**

We assessed the presence of viral RNA in samples from environmental surfaces before and after UV LED irradiation in COVID-19 patient rooms after patient discharge.

**Results:**

We analyzed 216 environmental samples from 17 rooms: 2 from airborne infection isolation rooms (AIIRs) in the intensive care unit (ICU) and 15 from isolation rooms in the community treatment center (CTC). Severe acute respiratory syndrome coronavirus 2 (SARS-CoV-2) RNA was detected in 40 (18.5%) of 216 samples after patient discharge: 12 (33.3%) of 36 samples from AIIRs in the ICU, and 28 (15.6%) of 180 samples from isolation rooms in the CTC. In 1 AIIR, all samples were PCR negative after UV LED irradiation. In the CTC rooms, 14 (8.6%) of the 163 samples were PCR positive after UV LED irradiation. However, viable virus was not recovered from the culture of any of the PCR-positive samples.

**Conclusions:**

Although no viable virus was recovered, SARS-CoV-2 RNA was detected on various environmental surfaces. The use of a UV LED disinfection robot was effective in spacious areas such as an ICU, but its effects varied in small spaces like CTC rooms. These findings suggest that the UV LED robot may need enough space to disinfect rooms without recontamination by machine wheels or insufficient disinfection by shadowing.

Severe acute respiratory syndrome coronavirus 2 (SARS-CoV-2) widely contaminates the patient environment.^1^ Manual cleaning using chemical disinfectants puts the cleaning staff at risk by exposing them to the virus, and it might also be insufficient depending on the type of ward and cleaning personnel. Furthermore, reducing the time for complete disinfection of patient rooms after patient discharge is critical for the efficient use of medical resources when several patients require healthcare simultaneously. Therefore, medical facilities consider no-touch environmental cleaning and disinfection methods, such as robot-assisted ultraviolet (UV) light irradiation and hydrogen peroxide vapor fumigation, useful to clean rooms used by coronavirus disease 2019 (COVID-19) patients and to prevent secondary transmission through fomites. Most studies evaluating no-touch technology have used UV irradiation as an adjuvant after terminal room cleaning because manual cleaning reduces the bioburden and increases the efficacy of the technology.^2–4^ In contrast, we explored the effectiveness of a UV LED robot without manual terminal cleaning of the room to minimize the chances of COVID-19 infection and cross contamination by the cleaning staff during a surge in COVID-19 patients. Studies have concluded that microorganisms, including coronavirus, are highly susceptible to UV inactivation.^5^ A recent study of SARS-CoV-2 reported that the virus is also rapidly inactivated by UV light-emitting diode (LED) irradiation.^6^ However, our study was an in vitro study, and no other studies have evaluated the effectiveness of UV LED disinfection in a real hospital setting. Therefore, we investigated the extent of environmental contamination and the effectiveness of a UV LED disinfection robot to decontaminate COVID-19 patient rooms.

## Methods

### Study design

Environmental samples were collected from COVID-19 patient rooms immediately after patient discharge (before UV) and after UV irradiation, and always before manual terminal cleaning. Medical staff involved in patient care and environmental sampling, UV LED robot experts, and microbiology laboratory personnel were involved and collaborated during this study. To implement robot disinfection, minimal information like room number, discharge time, and sampling time, was provided to the robot disinfection team. Information on patient demographics, medical conditions, and sampling sites were not disclosed to the robot disinfection team. Healthcare workers and the robot disinfection team were not informed about all interim environmental sampling results until the final analysis. Information regarding the patients’ medical condition and sampling sites was not disclosed to the microbiology laboratory.

Given the increasing number of COVID-19 cases, the Korean government has set up temporary facilities called community treatment centers (CTCs) to isolate asymptomatic or mild COVID-19 patients.^7^ CTCs only accept patients younger than 65 years, and mild or asymptomatic cases that do not require hospitalization. In Korea, COVID-19 confirmed cases were classified into asymptomatic, mild, severe, and very severe.^8^ Mild COVID-19 was defined as having no risk factors, tolerable symptoms such as fever <38°C with antipyretic drugs, and no need for oxygen therapy. Patients who needed oxygen therapy were classified as severe cases. Patients requiring noninvasive ventilation or invasive ventilation, extracorporeal membrane oxygenation or continuous renal replacement therapy were classified as very severe cases. This study analyzed data collected between April 2 and May 13, 2020, in 2 airborne infection isolation rooms (AIIRs) in the intensive care unit (ICU) for severe COVID-19 patients and 15 CTC rooms for mildly ill or asymptomatic patients.

The area of the COVID-19 zone in the ICU is 140 m^2^ and consists of 2 AIIRs without toilets, 2 anterooms, a corridor, and a doffing room. The airflow of the COVID-19 zone reached 15 air exchanges per hour. Following the participating hospital’s infection control guidelines, high-touch areas and floors were cleaned twice daily during patient hospitalization. Healthcare personnel wearing personal protective equipment cleaned high-touch surfaces with CaviWipes, and mopped the floor with 1,000 ppm sodium hypochlorite. The cleaning protocol for the COVID-19 zone in the ICU was designed by an internal committee for infection control, and the cleaning checklist and logs were audited by the committee. Each isolation room in the CTC is 20 m^2^ (including toilet) and has 2 single beds, 2 desks, and 2 chairs. The CTC facility did not have a negative-pressure air conditioning system and was naturally ventilated by opening the windows. The CTC rooms were only cleaned after patient discharge.

A UV LED robot (UVER-SR1, UVER Co, Gyeonggi-do, South Korea) was used for UV disinfection. The robot has a collaborative arm that can freely rotates with 8 multiple joints and reaches up to a maximum of 2,000 mm. The robot emits UV light with multiple wavelengths ranging from 340 to 385 nm (peak 365 nm). The UV LED light source is a chip on board arrayed with a wafer-level chip. Effective irradiation area of the UV LED module is 180 × 40 mm, and it emits a high power UV light with an intensity of up to 36,000 mW/cm^2^. We used 80% of full intensity in this study. The irradiation time was set to 30 s or 50 s per position, and the irradiation distance to 10 cm from the object. Sensors are embedded around the body of the robot to detect obstacles and walls, to calculate the robot’s arm extension or body movement without interference from objects, and to transfer information to the robot control software. Since the robot can also be operated manually, to minimize shadowing, the robot team experts adjusted the arm position and angle with a remote control while observing the robot.

This study was approved by the Institutional Review Board (IRB) of Korea University Ansan Hospital (IRB no. 2020AS0120).

### Sample collection

One AIIR (room 2) in the ICU and 15 isolation rooms in the CTC were sampled immediately after patient discharge (before UV decontamination). After the robot irradiated the room, environmental sampling was repeated. Sampling before and after UV disinfection were performed by the same medical staff. Another AIIR (room 1) of the ICU was not subjected to UV disinfection, and environmental samples were only collected after patient discharge. In room 2 of the ICU, additional samples were taken after manual terminal cleaning in addition to sampling before and after UV disinfection.

We used polyester-flocked oropharyngeal specimen-collection swabs moistened with viral transport medium (VTM) and swabbed in 3 directions. The surfaces sampled included floor, wall, high-touch areas, and toilet. The specimens were immediately transferred and analyzed in a biosafety level-3 laboratory using quantitative real-time reverse transcriptase (rRT)-polymerase chain reaction (PCR), nested PCR, and culture.

### rRT-PCR

Viral RNA was extracted using a commercial RNA isolation kit (Qiagen, Hilden Germany). Duplicate reactions were performed using the RNA extract, RNA-dependent RNA polymerase targeting primers/probes (forward: 5′GTGARATGGTCATGTGTGGCGG3′, reverse: 5′CARATGTTAAASACACTATTAGCATA3′, probe: FAM-5′CAGGTGGAACCTCATCAGGAGATGC3′-BHQ), and the TaqMan rRT-PCR one-step mix according to the protocol in QuantStudio software.

### Nested RT-PCR

Viral RNA was extracted using a commercial RNA isolation kit (Qiagen). cDNA synthesis was performed using the viral RNA extract, a target-specific reverse primer, and the SuperScript reverse transcriptase premix following the manufacturer’s protocol. The first PCR primers were ORF1a target forward: 5′TTCGGATGCTCGAACTGCACC3′, reverse: 5′CTTTACCAGCACGTGCTAGAAGG3′, spike protein target forward: 5′TTGGCAAAATTCAAGACTCACTTT3′, reverse: 5′TGTGGTTCATAAAAATTCCTTTGTG3′. The nested PCR primers were ORF1a target forward: 5′CTCGAACTGCACCTCATGG3′, reverse: 5′CAGAAGTTGTTATCGACATAGC3′, spike protein target forward: 5′TCAAGACTCACTTTCTTCCAC3′, reverse: 5′ATTTGAAACAAAGACACCTTCAC3′. Nested PCR products were analyzed using 1% agarose gel electrophoresis.

### Virus culture

Approximately 10% of the VTM specimen was subjected to a plaque assay. The plaque assay was conducted as follows: Vero cells plated 12 hours earlier at 9×10^5^ cells per well in 6-well plates were inoculated with specimens diluted in phosphate-buffered saline. After a 1-hour incubation, cells were overlaid with 2 mL DMEM/F12 medium containing 0.6% oxoid agar and were further incubated at 37°C and 5% CO_2_ for 72 hours. The plates were stained with crystal violet to visualize plaques formed by viable replicating virus. Virus were purified by isolating the virus from the plaques before staining and using an amplification culture.

## Results

### Patient characteristics

Table 1 presents the patient characteristics and SARS-CoV-2 detection rate of each room before and after UV LED disinfection. At discharge, ICU patients were asymptomatic, and SARS-CoV-2 PCR was negative for both sputum and nasopharyngeal swabs. None of the patients at the CTC had comorbidities, and the median age was 29 years (range, 15–63). Only 1 patient (CTC 1) who stayed in CTC had pneumonia. Patient CTC 1 was classified as stable at the time of diagnosis, but pneumonia was detected on chest radiography performed after admission. Since fever and pneumonia worsened, the patient was transferred to the hospital 4 days later. Moreover, 7 patients in the CTC rooms were transferred to other facilities while SARS-CoV-2 virus shedding continued. The average stay duration at CTC rooms was 19 days (range, 2–45 days).

**Table 1.** Patient Demographics, Clinical Characteristics, and Detection of SARS-CoV-2 RNA in the Isolation Rooms Before and After UV LED Disinfection

							Nested PCR Positive/Tested Samples, No. (%)	
Variable	Age	Sex	Length of Stay	Comorbidity	PCR at Discharge^a^	Pneumonia	Before UV	After UV
ICU, airborne transmission isolation room								
ICU 1	71	F	27	HTN, DM, dyslipidemia, hypothyroidism	(−)	Yes	5/18 (27.8)	ND
ICU 2	76	M	35	Atrial fibrillation, s/p rectal cancer	(−)	Yes	7/18 (38.9)	0/18 (0)
CTC, single room								
CTC 1	40	M	4	No	(+)	Yes	2/12 (16.7)	3/7 (42.9)
CTC 2	22	F	45	No	(−)	No	3/12 (25.0)	3/8 (37.5)
CTC 3	30	F	26	No	(−)	No	2/12 (16.7)	4/8 (50.0)
CTC 4	24	M	33	No	(−)	No	7/12 (58.3)	2/8 (25.0)
CTC 5	23	M	25	No	(−)	No	0/12 (0)	0/12 (0)
CTC 6	37	F	19	No	(−)	No	1/12 (8.3)	0/12 (0)
CTC 7	35	F	35	No	(−)	No	2/12 (18.2)	0/12 (0)
CTC 8	32	M	12	No	(−)	No	0/12 (0)	0/12 (0)
CTC 9	29	M	32	No	(+)	No	2/12 (16.7)	0/12 (0)
CTC 10	22	M	27	No	(+)	No	1/12 (8.3)	0/12 (0)
CTC 11	33	F	19	No	(+)	No	2/12 (16.7)	0/12 (0)
CTC 12	22	F	40	No	(+)	No	0/12 (0)	0/12 (0)
CTC 13	63	M	9	No	(+)	No	2/12 (16.7)	1/12 (8.3)
CTC 14	15	M	8	No	(−)	No	2/12 (16.7)	0/12 (0)
CTC 15	26	M	15	No	(+)	No	2/12 (16.7)	1/12 (8.3)

Note. SARS-CoV-2, severe acute respiratory syndrome coronavirus 2; RNA, ribonucleic acid; UV, ultraviolet; LED, light-emitting diode; PCR, polymerase chain reaction; ICU, intensive care unit; CTC, community treatment center; F, female; M, male; HTN, hypertension; DM, diabetes mellitus; ND, not done.

a It was considered positive when the Ct values of all genes were <40 cycles.

### Effect of UV LED disinfection by room

In addition to rRT-PCR, we used nested RT-PCR targeting 2 genes to increase detection sensitivity. SARS-CoV-2 RNA was undetectable by rRT-PCR but was detected by nested PCR. SARS-CoV-2 RNA was detected in 40 (18.5%) of 216 environmental samples after patient discharge. Viral RNA was detected in 12 (33.3%) of 36 samples from AIIRs in the ICU, and 28 (15.6%) of 180 samples from the CTC rooms. However, no viable virus was recovered from the culture of PCR-positive samples. The total disinfection time of each room with the UV LED robot, preset irradiation time per position, and irradiation distance are listed in Supplementary Table 1 (online). The time spent on disinfection varied according to room size, items, and arrangement of each room. For AIIR in the ICU, UV LED irradiation took 35 minutes; each CTC room took a median of 37 minutes and showed a difference of up to 40 minutes (range, 30–70).

In 1 ICU room where UV LED disinfection was performed, 7 of the 18 sites were PCR positive, and all turned negative after UV irradiation. In 3 CTC rooms (CTCs 5, 8, and 12), all sites were PCR negative before UV irradiation, and there was no change after UV disinfection. In 6 CTC rooms (CTCs 6, 7, 9, 10, 11, and 14), all contaminated sites turned negative after UV irradiation. In 3 rooms (CTCs 1, 2, and 3), viral RNA detection increased or remained unchanged, and 3 rooms (CTCs 4, 13, and 15) showed poor disinfection effect.

### Surface contamination, changes after UV LED irradiation, and terminal cleaning in AIIRs in the ICU

Before UV LED irradiation, SARS-CoV-2 was detected in 5 of 18 (27.8%) and 7 of 18 (38.9%) sampling sites in ICU rooms 1 and 2, respectively (Table 2). PCR-positive surfaces included frequently touched surfaces in the patient room and fixtures in the anteroom and doffing room, door windows, and floors.

**Table 2.** Detection of SARS-CoV-2 RNA in Airborne Infection Isolation Rooms After Discharge of a Severely Ill COVID-19 Patient, Before UV LED Disinfection, After UV LED Disinfection, and Terminal Cleaning

Variable	Room 1	Room 2		
		Before UV	After UV	After Terminal Cleaning
Isolation room				
Bed sheet	(−)	ORF 1a(+)	(−)	(−)
Bed side rail	(−)	(−)	(−)	(−)
Bed table	Spike (+)	(−)	(−)	(−)
Bed table 2	ND	(−)	(−)	(−)
Call bell	(−)	Spike (+)	(−)	(−)
Bedside wall	ND	(−)	(−)	(−)
Shelf	ND	(−)	(−)	(−)
Monitor	(−)	Spike (+)	(−)	(−)
Thermometer	(−)	ND	ND	ND
Floor	(−)	Spike (+)	(−)	(−)
IV pole	(−)	Spike (+)	(−)	(−)
Door window, room side	ND	(−)	(−)	(−)
Door window, anteroom side	ND	(−)	(−)	(−)
Air outlet fan	(−)	(−)	(−)	ORF 1a(+)
Anteroom				
Floor	Spike (+)	(−)	(−)	(−)
Desk	(−)	Spike (+)	(−)	(−)
Keyboard	Spike (+)	(−)	(−)	(−)
Door window, anteroom side	ND	Spike (+)	(−)	(−)
Door window, hallway side	ND	(−)	(−)	(−)
Hallway				
Floor	(−)	ND	ND	ND
Desk	(−)	ND	ND	ND
Notebook	(−)	ND	ND	ND
Doffing room				
Doorknob	Spike (+)	ND	ND	ND
Entrance wall	(−)	ND	ND	ND
Floor	Spike (+)	ND	ND	ND
Positive/tested samples, no. (%)	5/18 (27.8)	7/18 (38.9)	0/18 (0)	1/18 (5.6)

Note. SARS-CoV-2, severe acute respiratory syndrome coronavirus 2; RNA, ribonucleic acid; COVID-19, coronavirus disease 2019; UV, ultraviolet light; LED, light-emitting diode; ORF 1a, open reading frame 1a of SARS-CoV-2; Spike, spike protein of SARS-CoV-2; ND, not done.

In room 2 of the ICU, none of the additional 18 sampling sites were PCR positive after UV LED irradiation and terminal cleaning. However, after terminal cleaning, the air outlet fan site was PCR positive (Table 3).

**Table 3.** Detection of SARS-CoV-2 RNA in 15 Community Treatment Center Isolation Rooms Occupied by Patients With Mild COVID-19 by Swabbed Items and Ultraviolet (UV) Disinfection

Variable	Before UV		After UV	
	Samples Tested	SARS-CoV-2 Detected, No. (%)	Samples Tested	SARS-CoV-2 Detected, No. (%)
Room				
Bed sheet	15	2 (13.3)	15	0 (0)
Desk	15	0 (0)	15	2 (13.3)
TV remote	15	4 (26.7)	15	2 (13.3)
TV	15	2 (13.3)	15	0 (0)
Floor	15	4 (26.7)	15	5 (33.3)
Doorknob	15	5 (33.3)	14	1 (7.1)
Pillow	15	2 (13.3)	15	2 (13.3)
Blanket	15	1 (6.7)	15	1 (6.7)
Toilet				
Doorknob, room side	15	2 (13.3)	11	0 (0)
Doorknob, toilet side	15	1 (6.7)	11	1 (9.1)
Floor	15	2 (13.3)	11	0 (0)
Toilet seat	15	3 (20.0)	11	0 (0)
Total	180	28 (15.6)	163	14 (8.6)

Note. SARS-CoV-2, severe acute respiratory syndrome coronavirus 2; RNA, ribonucleic acid; CTC, community treatment center; COVID-19, coronavirus disease 2019; UV, ultraviolet.

### Contamination of environment surfaces before and after UV LED irradiation in CTC isolation rooms by site

In CTC rooms, 28 of 180 samples (15.6%) were PCR-positive before UV LED irradiation, and 14 (8.6%) of 163 samples were PCR-positive afterward (Table 3). SARS-CoV-2 RNA was detected at all sites, except the desk, before UV disinfection, and the viral RNA detection rate was high on the doorknob of patient rooms (33.3%), the TV remote controllers (26.7%), and the floor (26.7%). After UV disinfection, the viral RNA detection rate increased on the desk (from 0% to 13.3%) and floor of patient rooms (from 26.7% to 33.3%) but decreased in the remaining sites. Supplementary Table 2 (online) summarizes the results for each room and site.

## Discussion

In addition to traditional environmental disinfection methods, studies incorporating no-touch disinfection technology, like UV light, are being actively conducted at hospital rooms occupied by patients with multidrug-resistant infections such as *Clostridioides difficile*, vancomycin-resistant enterococci, and carbapenem-resistant Enterobacteriaceae infections.^2–4,9–11^ However, there have been no reports on no-touch environmental disinfection of rooms occupied by COVID-19 patients. UV irradiation could be applied to mitigate COVID-19 transmission. In this study, we evaluated the disinfection effect of UV robots in an actual clinical setting.

Recent studies have shown that SARS-CoV-2 contaminates multiple surfaces in rooms occupied by patients with active infection.^1,12–14^ Moreover, a previous study showed that SARS-CoV-2 transmission by aerosol and fomites is plausible^15^ because the virus remains on surfaces that discharged patients frequently touched during their stay. A study reported that SARS-CoV-2 RNA was detected by rRT-PCR in an isolation ward 28 days after patient discharge.^16^ However, we could not confirm the presence of viable virus in this study. The detection of SARS-CoV-2 RNA in a COVID-19 patient-occupied ward by rRT-PCR was not linked to the presence of viable virus.^17^ Although several studies reported the detection of SARS-CoV-2 RNA by rRT-PCR on surfaces of the patient environment,^1,12–14^ isolating the virus from cultures has been rare.^18^ We could detect viral RNA only by nested RT-PCR, not by rRT-PCR, or culture.

The UV LED robot moves its wheels and arms to disinfect items in the room. Therefore, the UV LED disinfection time varies depending on the room size, room items, and room arrangement. The CTC rooms were smaller than the ICU rooms but usually messier. Therefore, even though the same irradiation time was set, the total disinfection time per room could vary by up to 40 minutes for CTC rooms, which reflects the degree of dirt and complexity in each room.

The AIIRs in the ICU were cleaned every day, while the CTC rooms were not cleaned until discharge. Nevertheless, environmental contamination in the ICU immediately after discharge was higher than in the CTC (33.3% vs 15.6%). We suspect that this result is due to differences in disease severity among patients. However, due to the small sample size, statistical validation, including adjustment of other variables, was not performed. Moreover, this observation is not in line with the results of previous studies^14,19^ showing no association between virus shedding or environmental contamination and symptoms. Therefore, further studies with a larger sample are needed.

Although we did not perform air sampling, 1 swab from the air exhaust outlet tested positive. This has also been reported in a previous study,^1^ suggesting that small virus-laden droplets are carried by the airflow and accumulate in vents. Reviewing the irradiation record of the UV LED robot, we found that the outlet fan was not irradiated. Moreover, the outlet fan was not included in the terminal cleaning. We did not swab all the surfaces of the outlet fan to obtain PCR samples. Therefore, even though the PCR was negative both before and after UV irradiation, it is more reasonable to assume that there was some contamination from the beginning that was not removed during the UV irradiation and terminal cleaning processes. Thereafter, we changed the hospital’s protocol to include outlet fans in the terminal cleaning. Previous studies have predicted a low risk of transmission from contaminated shoes due to negative PCR results in the anteroom and corridor.^1^ In contrast, in this study, PCR test results were positive in the anteroom and doffing room floors of the ICU.

Although evaluated in only one room, UV LED disinfection in the ICU achieved adequate disinfection levels despite extensive environmental contamination. However, the effect of UV LEDs in CTC rooms varied for several reasons. The CTC room is narrower than the ICU, hence, the space in which the UV LED robot can move was limited. For example, after UV irradiation, the contaminated portion of the floors of CTC rooms increased because the robot wheel contaminated while passing through the corridor and touched the floor of the patient room thereafter. In CTC rooms, the toilet floor was irradiated by extending the robot’s arm in a fixed space without the machine wheel entering. Hence, no virus was detected on the floor of the toilet after UV irradiation. Nevertheless, there was incomplete disinfection not explainable by contamination by the robot alone. In the cases of CTC1 and 4, viral RNA was detected after UV disinfection even on a desk that was not affected by the robot’s wheels. The desk was a space where specimens were handled. Therefore, we suspect that medical staff caused this contamination because they visited several CTC rooms to collect samples. During the process, medical staff transited through contaminated areas, such as corridors or elevators, and possibly recontaminated the desks where multiple manipulations are performed. Another explanation is the difference in cleaning strategies. Daily cleaning in the ICU includes getting rid of the trash and tidying up the room in addition to wiping the surfaces. Therefore, although we detected no difference in virus detection in the environment after discharge, the UV LED robot may have been helpful in effectively sterilizing the room. For the CTC rooms, we did not obtain any samples after terminal decontamination without UV disinfection, and could not compare UV LED with terminal cleaning.

Although the PCR test results were positive for several environmental surface samples, none of the positive specimens produced viable virus in culture. However, given that viral RNA of multiple target genes was detectable (CTC 1, Supplementary Table 2 online), we can cautiously assume that virus transmission through environmental surfaces might still be possible. This study took advantage of a unique situation to test the UV irradiation cleaning technology in a real clinical setting and assessed the performance of the technology.

This study has several limitations. First, the sample size was small, and some room areas were not included. Second, while the 3 participating research teams were blinded to each other before the final analysis, environmental sampling was conducted by the same person before and after UV. This is advantageous because the sampler avoided testing the same spots swabbed before UV swabbing again after UV. However, there could have been a potential bias because the process was not blinded. Third, the possibility that the same space was swabbed again before and after UV irradiation cannot be completely excluded. Although medical staff tried not to reswab the same area in each site, the process relied solely on memory as areas were not marked. Therefore, it is possible that the first swabbing may have physically removed the viral particles.

In conclusion, although viable virus was not recovered, SARS-CoV-2 RNA was detected on various environmental surfaces. The UV LED disinfection robot was effective in a spacious area such as an ICU, but its effects varied in small spaces such as CTC rooms.

## Supplementary material

For supplementary material accompanying this paper visit http://dx.doi.org/10.1017/ice.2021.95. click here to view supplementary material
