# Supplementary material for: Feasibility of ultraviolet light-emitting diode irradiation robot for terminal decontamination of coronavirus disease 2019 (COVID-19) patient rooms
Source: Infect Control Hosp Epidemiol. 2021 Mar 9:1–6. doi: 10.1017/ice.2021.95 (PMC8160490; doi:10.1017/ice.2021.95)
Supplement: Supplementary file 1 [file S0899823X21000957sup001.docx]

Supplementary Table 1. Total time spent on sterilization of each room with the UV LED robot, pre-set irradiation time per position, and irradiation distance

|  | | Total disinfection time | Pre-set irradiation time | Pre-set irradiation distance |
| --- | --- | --- | --- | --- |
| ICU, airborne transmission isolation room | | | | |
|  | ICU 1 | ND | ND | ND |
|  | ICU 2 | 35 min | 30 s | 10 cm |
| CTC, single room | | | | |
|  | CTC 1 | 60 min | 50 s | 10 cm |
|  | CTC 2 | 40 min | 50 s | 10 cm |
|  | CTC 3 | 35 min | 50 s | 10 cm |
|  | CTC 4 | 30 min | 50 s | 10 cm |
|  | CTC 5 | 35 min | 50 s | 10 cm |
|  | CTC 6 | 30 min | 50 s | 10 cm |
|  | CTC 7 | 30 min | 50 s | 10 cm |
|  | CTC 8 | 40 min | 50 s | 10 cm |
|  | CTC 9 | 70 min | 50 s | 10 cm |
|  | CTC 10 | 40 min | 50 s | 10 cm |
|  | CTC 11 | 40 min | 50 s | 10 cm |
|  | CTC 12 | 50 min | 50 s | 10 cm |
|  | CTC 13 | 40 min | 50 s | 10 cm |
|  | CTC 14 | 35 min | 50 s | 10 cm |
|  | CTC 15 | 35 min | 50 s | 10 cm |

UV, ultraviolet light ; LED, light-emitting diode;; ICU, intensive care unit; CTC, community treatment center

Supplementary Table 2. Detection of SARS-CoV-2 RNA in CTC isolation rooms before and after UV LED disinfection

|  | CTC 1 | CTC 2 | CTC 3 | CTC 4 | CTC 5 | CTC 6 | CTC 7 | CTC 8 | CTC 9 | CTC 10 | CTC 11 | CTC 12 | CTC 13 | CTC 14 | CTC 15 |
| --- | --- | --- | --- | --- | --- | --- | --- | --- | --- | --- | --- | --- | --- | --- | --- |
| Room |  |  |  |  |  |  |  |  |  |  |  |  |  |  |  |
| Bed sheet | (-) → (-) | (-) → (-) | (-) → (-) | spike(+) → (-) | (-) → (-) | (-) → (-) | (-) → (-) | (-) → (-) | (-) → (-) | (-) → (-) | (-) → (-) | (-) → (-) | spike(+) → (-) | (-) → (-) | (-) → (-) |
| Desk | (-) → ORF(+) | (-) → (-) | (-) → (-) | (-) →spike(+) | (-) → (-) | (-) → (-) | (-) → (-) | (-) → (-) | (-) → (-) | (-) → (-) | (-) → (-) | (-) → (-) | (-) → (-) | (-) → (-) | (-) → (-) |
| TV remote | ORF(+) → ORF(+), spike(+) | (-) → (-) | (-) → spike(+) | spike(+) → (-) | (-) → (-) | (-) → (-) | (-) → (-) | (-) → (-) | (-) → (-) | (-) → (-) | (-) → (-) | (-) → (-) | (-) → (-) | spike(+) → (-) | spike(+) → (-) |
| TV | (-) → (-) | (-) → (-) | (-) → (-) | spike(+) → (-) | (-) → (-) | (-) → (-) | ND →(-) | (-) → (-) | (-) → (-) | (-) → (-) | (-) → (-) | (-) → (-) | (-) → (-) | ORF(+) → (-) | (-) → (-) |
| Floor | (-) → ORF(+) | spike(+) → spike(+) | (-) → spike(+) | (-) → spike(+) | (-) → (-) | spike(+) → (-) | spike(+) → (-) | (-) → (-) | (-) → (-) | (-) → (-) | (-) → (-) | (-) → (-) | (-) → (-) | (-) → (-) | spike(+) → spike(+) |
| Doorknob | (-) → ND | spike(+) → spike(+) | (-) → (-) | spike(+) → (-) | (-) → (-) | (-) → (-) | ORF(+) → (-) | (-) → (-) | (-) → (-) | (-) → (-) | spike(+) → (-) | (-) → (-) | spike(+) → (-) | (-) → (-) | (-) → (-) |
| Pillow | (-) → (-) | (-) → spike(+) | spike(+) → spike(+) | ORF(+) → (-) | (-) → (-) | (-) →(-) | (-) → (-) | (-) → (-) | (-) → (-) | (-) → (-) | (-) → (-) | (-) → (-) | (-) → (-) | (-) → (-) | (-) → (-) |
| Blanket | (-) →(-) | (-) → (-) | (-)→ spike(+) | (-) → (-) | (-) →(-) | (-) → (-) | (-) → (-) | (-) → (-) | (-) → (-) | (-) → (-) | (-) → (-) | (-) → (-) | (-) → (-) | (-) → (-) | (-) → (-) |
| Toilet |  |  |  |  |  |  |  |  |  |  |  |  |  |  |  |
| Doorknob, room-side | ORF(+) → ND | (-) → ND | (-) → ND | (-) → ND | (-) → (-) | (-) → (-) | (-) → (-) | (-) → (-) | (-) → (-) | spike(+) → (-) | (-) → (-) | (-) → (-) | (-) → (-) | (-) → (-) | (-) → (-) |
| Doorknob, toilet-side | (-) → ND | (-) → ND | (-) → ND | spike(+) → ND | (-) → (-) | (-) → (-) | (-) → (-) | (-) → (-) | (-) → (-) | (-) → (-) | (-) → (-) | (-) → (-) | (-)→spike(+) | (-) → (-) | (-) → (-) |
| Floor | (-) → ND | spike(+) → ND | (-) →ND | (-) →ND | (-) → (-) | (-) → (-) | (-) → (-) | (-) → (-) | spike(+) →(-) | (-) → (-) | (-) → (-) | (-) → (-) | (-) → (-) | (-) → (-) | (-) → (-) |
| Toilet seat | (-) → ND | (-) → ND | spike(+) → ND | spike(+) → ND | (-) → (-) | (-) → (-) | (-) → (-) | (-) → (-) | (-) → (-) | spike(+) → (-) | (-) → (-) | (-) → (-) | (-) → (-) | (-) → (-) | (-) → (-) |

SARS-CoV-2, severe acute respiratory syndrome coronavirus 2; RNA, ribonucleic acid; UV, ultraviolet light ; LED, light-emitting diode; PCR, polymerase chain reaction; Ct, cyclic threshold; ICU, intensive care unit; CTC, community treatment center; ORF 1a, open reading frame 1a of SARS-CoV-2; spike, spike protein of SARS-CoV-2; ND, not done
